# New variant of Creutzfeldt-Jakob (vCJD) disease and other human prion diseases under epidemiological surveillance in Brazil

**DOI:** 10.1590/S1980-57642008DN10400003

**Published:** 2007

**Authors:** Vera Lúcia Gattás, Antonio Silva Lima Neto, George Santiago Dimech, Denise Mancini, Ligia Maria Cantarino, José Ricardo Pio Marins, Expedito José Albuquerque Luna

**Affiliations:** 1 Technical Manager for Emergent and Re-Emergent Infectious Diseases - GT-DER/CGDT/DEVEP/SVS.; 2 Professor of Medicine Course at University of Fortaleza – UNIFOR .; 3 Technical Consultant - GT-DER /CGDT/DEVEP/SVS.; 4 Technical Manager for Parasitic Diseases and Epidemic Outbreak Response, from the General Laboratories Management – GDPAS/CGLAB/DEVEP/SVS).; 5 Technical Advisor - Technical Advisory Group for Human Prion Diseases (GTA - Prions).; 6 General Coordinator for Transmissible Diseases - CGDT/DEVEP/SVS.; 7 Director of Epidemiological Surveillance Department - DEVEP/SVS.

**Keywords:** prion, Creutzfeldt-Jakob disease, new variant Creutzfeldt-Jakob disease, epidemiological surveillance, príon, doença Creutzfeldt-Jakob, nova variante da doença de Creutzfeldt-Jakob, vigilância epidemiológica

## Abstract

To increase the timeliness of detection of human cases of the new variant of Creutzfeldt-Jakob disease (vCJD) and to reduce the risk of transmission, the Brazilian Ministry of Health has established and standardized rules and control measures. These include the definition of criteria for suspect cases, reporting, monitoring, and control measures for illness prevention and transmission. Guidelines to be used by the team of health care staff were published and distributed to health workers. A detailed proposal for a simplified system of surveillance for prion diseases was developed and mandatory reporting introduced. Additional effort is necessary to increase vCJD case detection, thus making it necessary to establish a partnership with health care services for best identification of suspected cases and dissemination of information to all involved in the service dealing with vCJD investigation.

## Abstract (pt)

Com o objetivo de detectar de maneira oportuna casos humanos da nova variante da Doença de Creutzfeldt-Jakob (vDCJ) e de reduzir o risco de transmissão da doença, o Ministério da Saúde (MS) vem estabelecendo e padronizando normas e medidas de controle. Estas incluem critérios de suspeita diagnóstica, notificação, monitoramento e medidas de prevenção da doença e seus mecanismos de transmissão. Procedimentos a serem adotados pela área de assistência à saúde também foram produzidos e divulgados. Desenvolveu-se uma proposta detalhada do Sistema Simplificado de Vigilância Epidemiológica das Doenças Priônicas, estabelecendo a compulsoriedade de sua notificação. Muito esforço ainda será necessário para efetiva e rotineira captação da ocorrência de um caso suspeito de vDCJ, por isso faz-se necessário o estabelecimento de uma parceria com os profissionais da assistência por serem estes os que têm a capacidade de identificar suspeitos e remeter a informação a todos aqueles que devem conhecê-la.

In view of the recent emergence of the new variant of Creutzfeldt-Jakob Disease (vCJD) in humans, along with its social and economic repercussions, the Brazilian Ministry of Health devised specific activities directed toward prevention of CJD in 2001, by commissioning a Task Force with the following purposes: to produce a report on CJD, to standardize the criteria for suspect cases, to standardize notification and monitoring, to suggest measures to reduce the risk of disease transmission within the country through health related products and procedure adopted by health care services, and to provide useful information for the institutions concerned, as well as for the community.^1^ To this end, resolutions were published adopting control measures, which have been constantly updated in the light of the latest scientific knowledge.^2^ The range of health related products based on raw materials extracted from animals, including bovine, sheep, goat and buffalo species, in addition to wild ruminants is broad. Materials derived from ruminants are used as components in the production of medicines, cosmetics and other health related products.^3^

Examples of health related products made from materials of animal origin include: heparin, glycerine, griseofulvin, insulin, magnesium stearate, stearin and polysorbate. For import of such products, ANVISA (the National Health Regulatory Agency) has established several legal requirements based on the potential degree of infectivity of animal tissue being imported and the risk attributed to the country of origin, defined by the number of infected cases identified in the region.^3^

ANVISA has published two resolutions: RDC 3O5, of November 14, 2002 and RDC 68 dated March 28, 2003.4 The latter establishes conditions for importing, trading, and exposure to consumption of these products while the second measure clarifies rules and procedures concerning the proper processing of materials used in patients with clinical suspicion of CJD or vCJD; biosafety procedures for handling of patients, samples and other materials potentially contaminated with CJD or vCJD agent; procedures for the handling of corpses; relative infectivity of the tissues and the body fluids of animals, as well as definitions for those areas running a geographic risk of transmission. Information on these measures is available at: http://www.gov.br/vacalouca/index.htm.

In this same period, a few possible suspect CJD cases were reported to the Health Surveillance Secretariat/Ministry of Health (SVS/MS). At this time however, there was no surveillance protocol in place. Therefore, in December 2004, the Technical Advisory Group for Prion Diseases (GTA – Prions) – was commissioned comprising members of the “National Health Regulatory Agency” (ANVISA), and the Brazilian association of neurologists (Academia Brasileira de Neurologia). GTA-Prions developed a detailed proposal for the establishment of the “Simplified System for the Surveillance of Prion Diseases”. The implementation of the system is currently directed towards the timely discovery of possible cases of Creutzfeldt-Jakob (CJD) Disease and its variant (vCJD); aiming to reduce under-reporting of prion diseases in Brazil; to better understand the epidemiological profile of this illness throughout the country; to enable epidemiological, clinical and laboratory investigation of reported cases; to adopt individual and collective protective measures upon detection of new cases of such diseases; and to deploy possible prevention and control measures in the event of cases being identified within Brazilian territory.

At the beginning of 2005, the Technical Advisory Group for Human Prion Diseases (GTA-Prions) put forward the following components of the surveillance system: a notification form with case definitions of the clinical categories of prion diseases; a protocol for epidemiological, clinical and laboratory investigation of notified cases; the description of epidemiological, clinical and laboratorial information flows within the system; the definition of the reference clinics where the system will be implemented, and the identification of laboratories that are equipped to carry out the tests proposed in the protocol.

The National Seminar on Human Prion Diseases was held on June 8, 2005, with the participation of members of the Ministry of Agriculture, proposed surveillance actions for the Bovine Spongiform Encephalopathies, (BSE) and had the main objective of submitting the surveillance system proposal to neurologists practicing at state level and to the states’ epidemiological surveillance units. In August the same year, a meeting was held by the GTA-Prion (TAG) to incorporate suggestions presented at the seminar.

On July 07, 2005, the Ministry of Health classified CJD as a disease requiring mandatory notification through Regulation No. 33/2005, making the Simplified System of Epidemiological Surveillance of Human Prion Diseases in Brazil official.^5^ The inclusion of the disease onto the lists of diseases for which suspect cases must be notified to health surveillance services to allow further mandatory investigation, raised awareness of the disease to health authorities who then began reporting cases in many Brazilian states.

From August 2005 through December 2006, 30 suspected cases of CJD were reported to SVS/MS, in 12 Brazilian states.

The system still has some limitations, mainly concerning laboratory diagnosis and the very nature of Human Prion Diseases.^5^ The complexity of some of the required procedures (including biosafety rules), and consequently the difficulty in establishing a steady flow of samples can delay definitive diagnosis, and compromise the outcome of epidemiological investigations.

Despite the difficulties outlined, the state surveillance services have successfully adapted their routines during investigations of suspected cases of prion disease and have managed, with the assistance of reference laboratories and neurology services involved, to notify a number several of cases given the rareness of the disease and unprecedented nature of the proposal.

With regard to laboratory surveillance, collaboration amongst research centers has been pursued, to devise possible, often highly complex, methodologies. In this context, detection of the 14-3-3 protein achieved by the Laboratory of Hospital das Clínicas/School of Medicine of São Paulo University (HCFMUSP), has greatly aided diagnosis of the sporadic form of the disease, currently representing the sole parametric parameter included in the criteria for probable diagnosis of vCJD, and presents greater than or equal sensitivity and specificity than typical electroencephalogram. In spite of current advances in diagnosing the disease, it is recommended that other tests available for detection should be used in conjunction.

Molecular diagnosis is being carried out by the Ludwig Institute, through purification of the DNA obtained after leukocyte separation. Direct sequencing is performed using High Resolution Liquid Chromatography (DHPLC). Polymorphism studies and mutation research in the human prion gene (PRNP) can be used as an aid to diagnose CJD, whereby researchers have associated the occurrence of polymorphisms at codon 129 and protease resistant chemical-physical properties of cerebral prion protein (PrP^Sc^) with the disease. Two PrPSc strains have also been reported, namely Types 1 and 2, along with three possible genotypes at codon 129 (Methione homozygous (MM), valine homozygous (VV) and heterozygous], responsible for 6 sub-types of the disease. The majority of sporadic CJD cases are MM1 or MV1.

Concerning histopathological diagnoses, sending of material to Rio de Janeiro Federal University laboratory is in place, where macroscopic studies of the brain, frontal lobe fragments, basal ganglia, temporal lobe, occipital lobe, mesencephalon, bridge, bulb and cerebellum are being carried out. The diagnosis is considered positive for CJD when the following 3 pathognomic characteristics of the disease are identified: Spongiosis, neuronal loss and gliosis. The microscopic aspect reveals spongiosis of the cortex due to reactive astrocytic gliosis, in proportion to the degree of neuronal loss.

Constant improvements in surveillance activities are aimed primarily at increasing the sensitivity of the system for early detection of vCJD cases, in view of the burden these place on the Brazilian health system. The decision to include other prion disease in the system stems from the syndromic features displayed by these pathologies, particularly in their initial stages, which make the devising of a notification form exclusively for suspected cases of the new variant unrealistic. Thus, all prion diseases are reported. At a later stage, cases not meeting the criteria for vCJD are excluded and may or may not call for control responses, in addition to the natural interest for clinical and epidemiological research.

The epidemiological notification form and flow of delivery of samples to the laboratory are shown below.
